# Active Aging: Social Entrepreneuring in Local Communities of Five European Countries

**DOI:** 10.3390/ijerph17072440

**Published:** 2020-04-03

**Authors:** Marco Socci, David Clarke, Andrea Principi

**Affiliations:** 1 Centre for Socio-Economic Research on Aging, IRCCS INRCA—National Institute of Health and Science on Aging, Via Santa Margherita 5, 60124 Ancona, Italy; a.principi@inrca.it; 2 University Centre Shrewsbury, University of Chester, Shrewsbury SY38HQ Shropshire, England; clarke.d@chester.ac.uk

**Keywords:** active aging, senior social entrepreneuring, social innovation, capacity building, social needs

## Abstract

Building on the active aging framework, the aim of this study, carried out between 2016 and 2018, is to analyze concrete experiences of older individuals acting as key players of social change in six local communities of five European countries (Bulgaria, Denmark, England, France, Spain). The 19 seniors involved in the study, according to social contexts, individual past experiences, knowledge, and motivations, acted as senior social entrepreneurs, trying to build a pathway towards social solutions for unmet social problems they detected in local communities. Data were collected via templates and questionnaires and analyzed using the thematic analysis. The results highlighted that the 16 local initiatives created by seniors concerned social problems such as food waste, social isolation, multicultural integration, etc. The social solutions implemented by seniors seemed to have the potential to produce social value and, to different degrees, encouraging results and impact. Since this “social experiment” provided evidence that senior social entrepreneuring could be a driver to solve societal problems, policy makers should sustain the spread of both social entrepreneurial mindset and practices at the European level, for catalyzing the active potential of older people for the benefit of European local communities.

## 1. Introduction

Older people are increasingly seen as useful resources to properly address important contemporary societal challenges [1], as population aging has consequences in many fields, at both European and national level. Thus, to stimulate and to monitor the active participation and the contribution of older people to society (e.g., in the labor market, in volunteering, informal care provision, political participation) has been a European policy aim for several years [2,3].

Active contributions of older people would be useful both to solve societal challenges, as well as to older individuals themselves, since active old agers benefit from aspects such as increased satisfaction with life, motivation, quality of life, and improved physical and mental health [2,4]. 

The above is in line with the concept of active aging, which has been the subject of extensive research in the last two decades, and has became an overarching goal of European policies for facing and responding to the challenges of population aging. However, active aging policies have often been dominated by a narrow economic or productivist approach, focusing on the labor market participation of older adults/people, prioritizing the extension of working life (e.g., through reforms aimed to increase the retirement age and abolishing early retirement opportunities, thus delaying retirement) and, therefore, overlooking other active aging domains, as for example social engagement and volunteering [5]. Indeed, active aging is a broad concept, and in general, its approach focuses on encouraging the participation of older adults in society, far beyond their exclusive participation in the labor market. That is why active aging has been defined as “a process of optimizing opportunities for health, participation, and security in order to enhance quality of life as people age” [6]. What is interesting is that this definition emphasizes good health, inclusion, and “continued participation in social, economic, cultural, spiritual, and civic affairs, not just the ability to be physically active or to participate in the labor force” [5]. Thus, active aging emphasizes an holistic and life-course approach, including quality of life, physical and mental well-being, and social participation, leading to a “win-win” situation, where individuals (micro level), organizations (meso level), and society (macro level) benefit from the adoption and implementation of active aging policies, strategies, and initiatives. According to the above, active aging could be considered as an umbrella concept, including many paid and unpaid activities, among which are voluntary and community work. Research highlighted that voluntary work is one of the possible ways through which people can remain active as they age. Furthermore, even the activation of older adults in volunteering may benefit both individuals and society. For example, on the one hand, the engagement of older adults in voluntary work help them not only to stay active, but even to enjoy social recognition and inclusion, contributing to their social, physical, and mental well-being. On the other hand, the involvement in volunteering activities (e.g., social care, recreational and local community work), may contribute to meeting social needs in local communities, thus having beneficial effects even for the society [7,8]. In this context, volunteering, by contributing in meeting social needs, could be a useful resource for addressing societal challenges, even in local communities [9]. In addressing societal challenges, volunteers can collaboratively and actively work toward meaningful projects, including commitments to changing social and community practices, and this could lead to a learning by doing process on how to effectively address societal challenges, offering transformative opportunities for both volunteers and the community [9].

An important social challenge is the shrinking availability of public funds to solve societal problems. For instance, the 2008 economic crisis is still having repercussions on national and local governments in terms of service provision [10]. This happens not only in Mediterranean or Eastern European countries, where the role of the state has always been marginal in this respect [11], but also in countries such as Sweden and the Netherlands, where traditionally citizens could count on high standard and quality services guaranteed by the state [12,13]. 

In the perspective of this study, under the active aging framework, social entrepreneurship as a concept is strongly oriented to address societal challenges, and deserves specific attention.

Social entrepreneurship is considered as an important tool to tackle unmet social challenges, driving social change [14]. It is often viewed as a means to alleviate social problems, catalyze social transformation, and create and sustain social value [15]. Even if most of the available definitions of social entrepreneurship focus mostly on “social” as the outcome (the end–[14]), some definitions focus also on social entrepreneuring as a process leading to social solutions. For example, social entrepreneuring needs to operate through clustering and networks [16]. It concerns the use of resource combinations to pursue opportunities aimed at the creation of organizations and/or practices that yield and sustain social benefits [17], for example, opportunities to create social value by stimulating social change or meeting social needs. When viewed as a process, social entrepreneurship involves the offering of services and products, and can also refer to the creation of new organizations [17], new ventures, or managing existing organizations in a different manner for enhancing social wealth. Social entrepreneurs are characterized by leadership skills and a passion to realize their vision of social change they want to achieve, to take risks to do so, and are creative [18], while throughput factors of social entrepreneuring are entrepreneurial orientation, critical thinking skills, and networking. Connectedness is the main key to success for social entrepreneurship, through which people become co-owners of the learning processes taking place [19]. 

**Figure 1:**
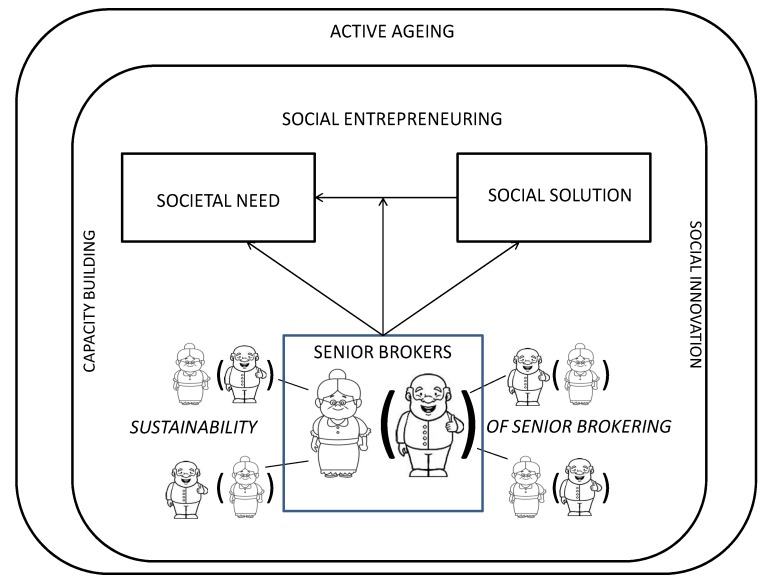
Senior social entrepreneuring: conceptual framework.

While the concept of social entrepreneuring is adopted as the fundamental element of the conceptual framework employed in this study (Figure 1), the latter includes also elements of the concepts of social innovation and capacity building, which are strongly connected to social entrepreneuring in the sense that they can be of help in creating social entrepreneuring [14,15]. 

Social entrepreneuring is connected to social innovation, since social entrepreneurs are searching for innovative solutions to meet new/unmet societal challenges and needs.

In the literature, the definitions of social innovation vary [20], however, there is a general agreement on key-characteristics of this concept. Social innovation is broadly meant as an intentional, purposeful new configuration of social practices realized through an integration of various constellations of stakeholders, in order to solve social problems or to satisfy needs better than would be possible on the basis of established practices. Social innovation is strongly encouraged at the European policy level, since it makes it possible to find new (and/or better) ways to solve social problems and/or needs [21]. Innovations are intended to be social both in their ends and their means, i.e., new ideas and solutions that simultaneously meet social needs and create new forms of social relationships or collaborations [22]. For being recognized as socially innovative, an initiative has to have certain characteristics, or meet certain criteria. 

According to some scholars [23], social innovation has to respond to the following main criteria: (1) relevance and consistency of the social problem should be well defined; (2) the innovativeness of the solution should be well defined and distinct from other existing solutions; (3) the capacity to develop, implement, and maintain the initiative should be clearly recognizable; (4) the initiative should generate an impact, and this impact should be measurable; (5) the innovation process should be open to stakeholder participation—by users in particular—in problem definition and solution development. The more an initiative adheres to these criteria, the more it is socially innovative. 

Like the concept of social innovation, capacity building also has many elements that are useful to develop social entrepreneurship [24]. Capacity building, meant as building abilities, relationships, and values to improve performances and to achieve objectives, also implies changes. Since it enhances cooperation between different groups of society, capacity building is meant as a catalyst and a constant fuel for a process of change [25]. It concerns empowerment and community development, raising people’s knowledge, awareness, and skills to use their own capacity to solve social problems. So capacity building concerns the shift from passivity to becoming active participants in a process of community change [26]. Connectedness is a main principle for capacity building, in which partnerships stimulate joined up working, resulting in improved knowledge and skills. In this way, partners can know each other’s views and find a common solution by extending their individual visions, thus achieving more by collaborating than by working alone [27]. A useful approach to capacity building is to create contacts between organizations or groups of people who might otherwise have little or no working relationship [28]. 

From the above it could be argued that in order to implement social changes through social entrepreneurship, to include social innovation and capacity building elements in the main framework would be essential. 

As can be observed in Figure 1, the background element is represented by the active aging perspective, and more precisely by its specific domains of volunteering and social engagement. The main aim of this study is to monitor concrete experiences of older individuals as key-players of social changes in European local communities, building on the concept of social entrepreneurship, and considering elements of social innovation and capacity building (Figure 1). 

In the perspective of this study, older people would represent the point of convergence between a societal need identified in a given local community, and the social solution for that need through social change. 

*“Senior social brokers”* are intended as the social entrepreneurs who are supposed to: identify an unmet societal need in their local community; think to the social solution and try to practically plan and develop their ideas. This should be done by building and implementing a pathway from the need to the solution, thus creating social change, even inspiring and involving other potential senior social brokers. A broker is a mediator, someone who talks to others and makes arrangements for deals and agreements; according to this, he/she tries to build a partnership with other senior social brokers and/or other stakeholders for implementing the solution identified for solving local unmet social needs, thus activating a process of social change in local communities.

*Societal need:* represented by any kind of possible social need identified by the senior brokers in the local community, which currently is not addressed by any public or private or third sector organization, and so needs to be addressed, since some kind of intervention/solution is required. It could be in the field of e.g., food, health, job, security, transports, social integration, etc.

*Social solution*: the idea, the initiative, identified and to be socially created by the seniors to be implemented in local communities, with the aim to address the identified social need.

*Social change*: the change activated through the implementation of the social solutions created and developed by seniors to address the identified unmet social needs in local communities.

A strength of this study is that it relies on useful elements of different concepts as described above, to concretely build answers to respond to social problems. There have been few experiences of this. Previous examples are represented by collections and evaluations of European socially innovative practices for active and healthy aging [23], for long-term care provision [29], for extending working lives [30], and pilot projects to empower older people to contribute to improving services for themselves [28]. However, none of them employed the social entrepreneuring approach. Central questions to be answered by this study are: is senior social entrepreneurship a useful way of solving societal problems? What can be done to promote senior social entrepreneurship in local communities? What are the main barriers to overcome? These are interesting questions from a policy perspective, especially with the current climate of shrinking economic resources that characterize European local communities. An interesting aspect of this study is that it concerns local communities from five European countries representing a range of different welfare regimes: Bulgaria (Post-communist); Denmark (Social-democratic); England (Liberal), France (Conservative) and Spain (Mediterranean) [31,32,33]. It is important to highlight that the share of volunteering in older age among the population is different in the countries under study. In the Social-democratic and Liberal regimes (Denmark and England) it is high, while volunteering in older age is less widespread in the Conservative one (France), and this share is low in the Mediterranean (Spain) and Post-Communist (Bulgaria) welfare regimes [7]. This situation is also the result of the interaction of several aspects at the macro level, e.g., the regimes’ characteristics (including culture, motivation, and aspirations of individuals), their different welfare mix, public policies, and laws for promoting volunteering in older age. For example, in the Social-democratic regime, no laws or policies for promoting volunteering at any age are in place, even though, as already mentioned, volunteering rates in older age people are high, thus highlighting that high volunteering propensity can coexist with few efforts at the policy level. Even France (Conservative regime) does not has specific policies in place for involving older people in volunteering, even though in such country the participation rate in voluntary work in older age is “medium”. Conversely, in countries belonging to the Mediterranean and Post-Communist regimes, specific laws for promoting volunteering exist, but, as seen, in these contexts voluntary work in older age is less widespread (even though a trend of increase is observable). In the Liberal regime (England), the longstanding tradition of policies aimed at promoting volunteering in older age is at the basis of high volunteering rates of seniors that do not decrease among people aged 65 and over [7]. Even if this framework might in general affect the willingness of older people to engage in volunteering as senior social brokers, it is worth clarifying that this explorative study does not intend to explain senior social entrepreneurship in the five countries under study. Yet, it intends to provide the first results about social needs to be addressed and social solutions to be implemented in different European local communities. Due to the diversity of the six local communities under study (in terms of welfare regime, population size, etc.; see also below) we expect to observe differences. The conceptual framework discussed above is intended to represent a common model to implement in local communities with their own specificities, and so it would or should have different contents in terms of social needs, social solutions, and social changes, depending on the specific context.

## 2. Materials and Methods

The study, based on the EU Erasmus Plus international project “Senior Social Entrepreneuring”, aimed to develop the ability of seniors for creating, as social change brokers, new initiatives to address unmet social needs. It was carried out between 2016 and 2018 in six local communities of five European countries: Aarhus (Denmark), Paris and Pau (France), Sabadell (Spain), Shrewsbury (England) and Sofia (Bulgaria). Local communities differed from each other, being in countries with different welfare states, laws, and regulations, and also differed in terms of population: from less than 100,000 inhabitants of Shrewsbury and Pau, to between 200,000 and 300,000 inhabitants of Aarhus and Sabadell, up to over one million in Sofia, and over two million in Paris. These communities differ even in other aspects, such as, for example, the share of older people aged 65 and over—lower than 20% in Aarhus (14%), Sofia (17,5%), and Sabadell (18,5%), and higher than 20% in Shrewsbury (20,7%), Paris (21,8%), and Pau (22,2%)—the economic structure, and the supply of formal social and health services (e.g., more widespread in Aarhus and Paris, lower in Sofia). In this way it was possible to consider societal needs of very different social communities. 

### 2.1. Recruitment and Sample

The recruitment of seniors took place from October 2016 to March 2017. Seniors were selected through a purposive, non-probability technique [34]. Moreover, sampling criteria used by project partners to recruit senior brokers in the six local communities were flexible, so they have been recruited depending on what was perceived, in the given local community, to be the best way to involve them. This has meant in some cases to use one’s own local networks, with phone calls and e-mails (Aarhus, Paris, Pau, Sofia), to present the project in volunteer centers (Aarhus) or individually (Pau, Sofia), through the website (Sofia), or to use the University as a vehicle for promotion, advertising the project at events, via social media (Shrewsbury), personal contacts or contacting old members of committees of the organization and senior associations (Sabadell, Shrewsbury).

Meetings were organized by the project partners, with the seniors who expressed an interest in being involved in the project, to evaluate their motivations after having described more in-depth their supposed role. They were informed about the project aims, the main tasks expected by them (i.e., to identify and address a societal need, and trying to practically build the pathway to the solution for that need), the expected results, and the methodological steps to monitor their “senior brokers” experience (i.e., an “individual portfolio” where they could report their experience). All the seniors who accepted to be part of the study were selected. There were 19 (five in Sofia, four in Sabadell, three in Aarhus, three in Paris, three in Shrewsbury, one in Pau), eight men and 11 women, with a mean age of 64.3 years; most of them had a high educational level and a reasonably high-level professional background. At the recruitment stage, the majority of seniors were retired, even though five of them were employed and two were unemployed. Most of the seniors were already engaged in doing voluntary activities in their local communities (Table 1). Another six seniors (three in Shrewsbury, three in Sofia) were initially recruited, however, for different reasons (worsened health, problems to reconcile this activity with other tasks, death, decreased motivation), gave up on the project at a very initial stage of the activities, and were not included in Table 1.

### 2.2. Data Collection, Measuring Tools, and Data Analysis

Data collection took place from October 2016 until April 2018. Common tools for data collection were employed in the local communities. This concerned common templates to be used for: reporting the recruitment process; describing the recruited seniors (e.g., age, gender, educational level, work status, and reasons and motivations for participating in the project); drafting the minutes of the periodic meetings in local communities with senior brokers, in order to monitor progresses and to plan future activities. Moreover, a template for reporting the seniors’ experience (i.e., the “senior portfolio”) was to be used by each senior as a sort of “diary”, to “tell the story” they were building, from the identification of the social need to the solution for that need, in a step-by-step process (i.e., social need identified, plans to address the social need, possible difficulties encountered, possible alternative plans and steps, etc.), in line with the conceptual framework adopted. 

### 2.3. Measures

The project team analyzed the results also considering self-administered questionnaires filled-in by the seniors, in order to have the possibility to measure issues such as the extent to which elements belonging to the concepts of social entrepreneurship (i.e., aspects related to the description of their “story”), social innovation (questions on the innovativeness of the proposed social solution) and capacity building (questions on, e.g., practical usefulness of the capacity building seminars, as reflected in the implementation of the solutions), characterized their experiences as social entrepreneurs.

In terms of *social entrepreneurship*, the interest was to allow seniors to tell their “story” through the “portfolio”: experiences as social entrepreneurs from the identification of the social need in the local community, how they built (or tried to build) the pathway to the solution of that need.

To understand whether *social innovation* was a component of the pathway built by the seniors, we based our work mainly on Kesselring and colleagues [23], who identified five social innovation criteria: (1) relevance and consistency of social needs definition; (2) innovativeness and social acceptance of the solution; (3) openness of the innovation process (e.g., networking and stakeholder participation); (4) estimated impact of the solutions implemented; (5) potential capacity to implement, develop, and maintain the initiative. As in previous experiences [23,29], the evaluation concerning the level of social innovation (presence or not of each criterion, Y/N) in the initiatives implemented was carried out by the project team.

To analyze *capacity building*, we also gathered information about the effect of two international “capacity building international seminars”, the first in Pau (France) in May 2017, and the second in Sabadell (Spain) in November 2017, where the seniors from different local communities met in person to exchange their experiences and ideas about their local initiatives. We also based on interactions of seniors on a project web-tool specifically used for keeping them in contact.

Almost all the seniors could communicate in the English language. When needed, translation (written and oral) was provided by the local project partners. The documents from field texts produced during the project activities were analyzed using a thematic analysis technique [35]. Textual data were coded thematically according to topics, then, data were reduced and compiled in a framework matrix, using a spreadsheet to show data themes across columns and respondents by rows. A synthesis of respondent data for each theme was inserted into each cell. The main important themes were reported in the matrix, allowing comparisons across local communities. In particular, perceptions and information provided by seniors (e.g., via questionnaire answers and the “senior portfolio”) were grouped on the basis of commonalities identified among their significant statements, and, according to common statements, categories have been created, and themes emerged from them. The main themes that emerged were e.g., “motivations to participate”, “social unmet needs”, “pathway/social solution built”, “difficulties encountered”, “socially innovative aspects”, “capacity building”. 

The results will be reported in the following order: the pathway built by the seniors (social entrepreneurship), the innovativeness of this pathway (social innovation), and the usefulness of the interaction between the seniors (capacity building). 

## 3. Results

### 3.1. Social Entrepreneuring: Needs, Solutions, Successes, and Difficulties

All the seniors involved in the project in the six European local communities showed a high level of motivation for being part of it. *Motivations* ranged from the possibilities of cooperating with peers from abroad, of being useful to and to improve the community by producing social change, of being active after retirement, of sharing experience and knowledge by learning from each other, and of helping people in need. Thus, seniors highlighted both altruistic and self-expressive motivations for being engaged in project, and for carrying out voluntary work in their local communities acting as social entrepreneurs. The seniors involved in the project created 16 initiatives: five in Sofia, three both in Aarhus and Paris, two each in Sabadell and Shrewsbury, one in Pau. Five initiatives have been created and developed together by more than one senior (Table 2). Using a social entrepreneur approach, and based on specific characteristics of their local contexts and on their individual interests and motivations, seniors started the experience by detecting a range of *unmet social needs* to address in their local communities. The most common ones were social isolation and loneliness among older and disadvantaged people living in rural and urban areas (e.g., in Shrewsbury and Sofia) or in health care facilities (e.g., in Aarhus and Paris), and the lack of multicultural integration between migrants and native residents (e.g., in Aarhus and Pau). Other social needs identified by seniors were: food waste (in Shrewsbury), need to strengthen both social engagement of seniors and volunteering networks (e.g., in Aarhus and Paris), lack of entrepreneurial skills among both social companies and young people (in Sabadell), inadequate computer skills for senior health care workers (in Sofia). The social needs identified by seniors were related not only to their experience and interests, but even according to specific needs considered sensible and important in the various local communities. Some social needs were similar in local contexts, while others were detected only in one of them, thus emerging differences between local communities concerning this aspect.

After having identified the social needs in the local community, for creating the initiatives, seniors built an original *pathway* towards the *social solution* that was supposed to solve each need. For example, the lack of multicultural integration in the local community was a similar social need identified by seniors in Aarhus and Pau. However, in Aarhus, seniors organized informal “food events”, enhancing cultural and social cohesion among migrants and Danish people living in the community. In Pau, seniors really acted as social brokers. They put other local seniors in contact with asylum seekers, who implemented the initiative together. In particular, dyads of seniors and migrant asylum seekers were formed, contributing in this way to foster social inclusion of migrants with the local population. Social isolation and loneliness of disadvantaged groups of people were the most widespread societal needs detected by seniors in local communities. For tackling such needs in Aarhus and Paris, senior social entrepreneurs used music (i.e., performing respectively rock concerts and vocal concerts) for the benefit of older people living in health care facilities. Moreover, in the case of Aarhus, the senior social entrepreneur who launched this “artistic” initiative, involved disadvantaged people (with health problems) in playing music in nursing homes, as well as in informal settings, contributing in this way also to combat social isolation of these disadvantaged people, building with them a partnership for implementing the solution to such needs, acting as social brokers. Other solutions pursued by senior social entrepreneurs in this respect concerned the organization of workshops and conferences to stimulate social relationships/inclusion among retired people in Paris. Furthermore, in Sofia three senior brokers built networks of both older people and persons of different ages for strengthening social relationships and tackling social isolation of neighbors living in the same residential block, or of people living in a small village. For pursuing such goals, these Bulgarian senior social entrepreneurs promoted different initiatives: the establishment of a social center; activities for taking care of green spaces in the surroundings of their buildings; mutual help; and meetings among neighbors living in the same residential area. In Shrewsbury, for combating the isolation of people living in rural areas and the lack of local transport, seniors set up a community bus run by volunteers for allowing older and vulnerable people to be linked to health care facilities or other organizations for managing their personal needs. With the aim to strengthen volunteering networks and to empower volunteers, in Aarhus, seniors organized a meeting between volunteers living in an urban area with others living in a rural community, favoring mutual exchange and inspiration. In Shrewsbury, a project created by a senior social entrepreneur and managed in collaboration with more than 30 volunteers (mainly seniors), was aimed to combat food waste, collecting food safe to use but no longer marketable from supermarkets, and redistributing it to NGOs in the local community. For promoting active aging, in Paris, a senior created an international network of senior change brokers using an internet platform. Other initiatives had a strong link with the seniors’ professional background. For instance, in Sabadell, seniors, by exploiting their former experience and know-how as business managers and entrepreneurs, created solutions for promoting an entrepreneurial mentality among students of high schools, and for providing the needed business management skills to social entities (e.g., NGOs) working in the community. 

The role of *seniors as social mediators* in building and implementing their projects and initiatives emerged not only through their successful actions for involving other people (e.g., volunteers, older people, asylum seekers), but even considering their capacity of establishing collaborations and networks with many stakeholders. For example, for realizing the project to combat food waste in Shrewsbury, the senior broker organized meetings for presenting his idea to supermarket managers. Some of them supported his initiative, thus, through this mediation, the senior broker was able to establish a collaboration with five local supermarkets able to provide safe but no longer marketable food, as well as to reach 40 NGOs and charities to distribute this food. In Aarhus, the senior who conceived the idea of creating music events for tackling the social isolation of disadvantaged people was able to involve and to receive the support of the municipality, who offered a venue for playing music and helped the senior to organize concerts in several nursing homes in the city. In Pau, the initiative for promoting social integration of migrants had been created by the social broker by presenting the idea and then involving a local association helping migrants and refugees. In several other cases, the key mediating role of seniors social brokers emerged, for example by presenting their solutions to a range of different stakeholders (e.g., mayors, social and health professionals, public health and social services, NGOs, schools, etc.), and obtaining in many cases concrete support and help for implementing their initiatives. 

The majority of the solutions implemented by senior social entrepreneurs highlighted positive results and successes, in terms of beneficiaries and concrete *impact* on local communities. “Target groups” of initiatives had different socio-economic characteristics (e.g., retired or working persons, people with or without disabilities, migrants or native people, older people, and/or people of different age groups), lived in local communities with specific peculiarities (e.g., different number of inhabitants, different level of supply of social services, urban or rural areas), and the number of beneficiaries of the solutions created by seniors was quite high. For example, both of the local projects implemented in Shrewsbury reached hundreds of people of different age groups, with a significant proportion of people aged 50+. In seven initiatives created in different local communities (Aarhus, Paris, Pau, Sofia) the number of beneficiaries ranged from about 10 to 40 people, and among them the proportion of seniors (aged 50+) were fairly high, from about 40% to 60% of the total. The solutions created in Sabadell involved around 200 students and about 10 social entities working locally, even though only three of them accepted receiving support from senior social entrepreneurs. In the case of an initiative developed in Paris, the number of beneficiaries varies according to situations (e.g., spectators of concerts performed in nursing homes). Even though some initiatives were just at an initial and developmental phase (e.g., two projects in Sofia), overall, to different degrees, the local projects conceived by senior social entrepreneurs have started to make a difference for beneficiaries and their local communities, through activities able to provide an “answer” and solutions to unmet social needs, and to achieve, for example, the reduction of food waste, and the promotion of social inclusion of disadvantaged people (e.g., migrants, disabled people, etc.) in rural and urban areas. 

During the implementation process, most of the initiatives encountered some kind of *difficulties*, according to the specificity of the solutions pursued and the interaction with the specific environment. In Pau for example, it was a challenge to find migrants to participate in the project, probably due to diffidence and language barriers. In Sabadell, the main difficulty was to stimulate a change of mentality in social entities that, accustomed to specific routines, were often reluctant to change things, despite needing to improve the performance under several aspects. A difficulty in Paris that concerned the initiative “Breaking loneliness with music”, was the bureaucracy linked to ethical issues, this demonstrating how excessive bureaucracy can sometimes be counterproductive, when the “common good” would to be pursued in an informal and simple way. In several cases, difficulties were “transversal”, and similar across initiatives. For example, and not surprisingly, problems of funds (in Aarhus, in Paris, in Shrewsbury, and in Sofia); lack of time/problems in time management (in Paris, in Shrewsbury and in Sofia), especially for beneficiaries who were still working (e.g., in Sofia); logistic aspects/finding appropriate locations for implementing the initiatives (e.g., in Sofia); management and administrative issues (in Paris and in Shrewsbury). The latter was particularly frustrating for the seniors, since they were not experts on these issues and just wanted to concentrate on the core activity, however, especially when the initiatives grow fast involving a considerable number of users, these aspects become as important as the core activity itself. 

### 3.2. Social Innovation Elements 

The initiatives were found to be highly characterized by social innovation elements, especially in terms of relevance of the problem, innovativeness to respond to the social need identified, and openness of process in terms of stakeholder participation (practically all the solutions created by the seniors succeeded in activating stakeholder participation), and cooperation-combination of resources. Six initiatives (two in Aarhus and in Shrewsbury, one in Pau and in Sabadell) met all five social innovation criteria adopted in this study; six solutions (three in Sofia, two in Paris, and one in Sabadell) matched four criteria. The socially innovative criteria most difficult to demonstrate were the impact estimation of the initiative implemented in terms of type, scale, and measurable evidence, and the potential capacity to implement, develop, and maintain the initiative. The latter criterion included aspects such as knowledge, competences, management, and coordination [23]. This concerned in particular two local projects (in Aarhus and in Paris) that met three criteria and two initiatives (in Sofia) that were in line just with two social innovation criteria. From the point of view of the seniors, the *socially innovative aspects* most appreciated were related to the feeling that the initiatives they implemented to solve the identified social problems were better than other previous existing solutions.

### 3.3. Capacity Building Elements 

In terms of *capacity building*, the participation in two international capacity building seminars, and the related creation of an international network of senior brokers, positively influenced, inspired, and supported the work of seniors for implementing their initiatives in local communities. Seniors highlighted that exchanging views, information, and experiences with their peers from other countries allowed them to: see their local project from a different perspective; consider different approaches to deal with the challenges faced in implementing the initiatives; be inspired (even by ways/methods for involving people, e.g., volunteers); acquire more confidence and better understand the pathway to follow from the solution to its actual implementation; and to understand the challenges faced in other local communities. However, in some cases seniors would also have preferred to have more time for discussing the projects more in-depth, for providing and/or receiving additional constructive clues about the management of their pathway to the social solution for the identified social need. A group of seniors observed that attending to the international capacity building seminars, as well as having had the possibility to interact through a communication software made available by the project partnership, stimulated them into feeling part of an international community. 

Linked to the concept of capacity building, an important element of our study was the sustainability of senior brokering. This not in terms of sustainability of the initiatives created by seniors, which was a trait that we considered as part of the social innovation concept (i.e., part of the fifth criterion), but rather in terms of, beyond pursuing their initiatives, involving other seniors in activities of senior brokering, to make it sustainable, and to spread senior brokering in itself. Senior social entrepreneurs, to different degrees, and according to specificities of both their local communities and of the solutions created, put in place some activities in this respect. In several cases (e.g., in Aarhus and Sofia), these activities consisted of the organization of meetings with other seniors that may have been interested in social change brokering. In these meetings, the senior social entrepreneurs presented the solutions they implemented in local communities, with the aim to motivate other seniors in joining their current local projects, or encouraging them to develop their own (i.e., other, different) initiatives. In other cases (e.g., in Sofia) seniors presented their ideas/solutions to stakeholders (e.g., politicians) and to other people (e.g., friends, neighbors) for raising awareness about the added value for society, thanks to social brokering.

## 4. Discussion

Under the active aging framework—which implies a shift from a vision of older people from inactive and socially disengaged individuals in need, to active and resourced individuals with a positive potential to express [8]—the main aim of this study was to build a “social experiment” configurable as action research [36] to improve local communities. Seniors involved in the project acted as “social entrepreneurs”, doing informal voluntary work for the benefit of their community: they identified social needs in their local communities, and tried to build, according to their own individual experiences and knowledge, motivations and aspirations, the pathway towards the solution for those needs in a socially innovative and creative way. To answer to our main research question, senior social entrepreneuring seems like it could be a promising way to solve societal problems. Several social solutions have been created by seniors, built and put in place often involving other seniors and various stakeholders, and still running, thus contributing to social changes, in order to address important social needs in the local community, big or small, and the impact of this may have involved beneficiaries or people in general. Even if more data would be needed to confirm the real impact of those initiatives over time, a positive aspect is seen in the fact that the initiatives put in place allowed seniors to organize a social answer to specific social needs otherwise not socially addressed. Thus, the fact that now those specific needs started to be addressed thanks to the seniors as the main actors (to identify the need, to plan and implement the solution), could be considered a first positive factor in itself, even taking in consideration, at the local level, the number of beneficiaries of such initiatives. The latter seem also to have the potential to create social value with a very low economic outflow, and concerned for instance, efforts to fight social isolation, loneliness, and food waste, to improve work-related skills of older people, and to support multicultural integration and social engagement, which are recognized as crucial social needs [37,38,39,40].

Concerning the second research question about what can be done to promote senior social entrepreneuring in local communities, looking at the experiences of the seniors involved in the study, the first aspect may be to push their motivation, and/or to motivate them to act as senior social brokers. Indeed, all the seniors involved in the project showed a high level of intrinsic motivation by expressing various reasons for participating, which encompassed both altruistic and self-expressive forms of engagement through volunteering activities, acting as social entrepreneuers. In line with this, some older individuals with low motivation gave up soon after their recruitment in the project. So, a first message for policy making would be to try to involve older people by leveraging individual motivations and aspirations; to make it understand that it is possible to realize “social projects” in their preferred social field. This was also visible through the fact that, although the study was conducted in five European countries, by analyzing the initiatives, we did not find particular elements of senior social entrepreneuring linked to the kind of welfare regime in terms of social needs identified, or of ways to develop the initiative towards the solution, or of the level of social innovation. Instead, we found several elements of links with personal individual (past or current) life experiences of the seniors involved. For example, in Paris, a senior member of a choir developed a local project concerning the performance of choir concerts in health care facilities, while in Sabadell, former top managers provided managerial skills (to social enterprises and young students). In other cases, seniors created solutions to tackle local social needs without a link with their former professional career (e.g., “World Food Events” in Aarhus, “Shrewsbury Food Hub” in Shrewsbury). In these cases, they were guided by their passion, or by the challenge to start a completely new activity in their life. However, in some cases, the social needs detected were related to specific unmet needs considered “sensible” for the local communities where the seniors were living. For example, the “Market Drayton Community Enterprise” in Shrewsbuy is an initiative that has been created by a senior entrepreneur for tackling the social isolation of people living in rural areas due the lack of local transport service in that local community, due to cuts of funding made by the government. Even in Sofia, some initiatives have been conceived and implemented for combating social isolation, and for building networks among people living in the areas where seniors who created the solutions were living.

In terms of policy actions, it would be very important to provide opportunities for being useful in the community both to older people with a high level and a low level of resources. The experiences in the local communities under study demonstrated that it is relatively easy to involve older people with a high level of resources (i.e., the recruited seniors were mostly highly educated and qualified). This aspect is in line with the resource theory of volunteer work, according to which, having higher resources available in terms of socioeconomic status, including education, skills, and income, as well as health, facilitate volunteering [41]. However, less resourced older people could have higher motivation than other older people [42], and should be given the opportunities to develop their “social plans” through special attention at policy level. Another kind of social resource facilitating volunteering, according to the resource theory of volunteer work [43], is labor market participation. In this perspective, due to missing work-related social contacts, retired people may be disadvantaged in terms of volunteering opportunities, despite more time available [41]. Our study demonstrated that initiatives could be put in place both by employed and by retired older people, so that senior social entrepreneuring should be promoted both among employed people and retired ones.

In relation to our third research question regarding barriers to overcome, we found scarcity of funds to be a major challenge. This is no news, and policy makers should make an effort to guarantee sufficient funds to at least start the initiatives. Then, it would be possible to gradually reduce funds in time, until the initiative has settled and found the way to be self-sustainable, from the perspective of social innovation’s principles [23]. Lack of, or difficulties managing time to devote to the initiative due to concomitant (professional, family, or personal) tasks, has been another barrier. In light of this, policy efforts should be made to ensure that older people understand that social involvement may be flexible and may not necessarily concern a daily commitment, but, for instance, a few hours a week, in accordance with the needs of each senior. Other messages about barriers to overcome came from the analysis of social innovation and of the capacity building concepts. Regarding social innovation, overall, the social solutions created by the seniors showed a very good level of social innovation (according to the criteria employed in this study), and almost all of the initiatives succeeded in involving stakeholders, but this was not always an easy process, e.g., in Pau with migrants, in Sabadell with social entities. This means that policy efforts are needed to sensitize stakeholders on the need to networking for the common good. An additional difficulty encountered was that some initiatives evolved so much from the initial idea that it became too difficult to manage their growth and development, in terms of management and administrative issues. It is no surprise that seniors prefer to concentrate on the activities they enjoy [44], which is the core activity. While ensuring funds, the policy level should ensure that part of the fund is provided for management and administrative issues, e.g., in terms of training on these issues.

A further policy message, is that, as the international capacity building seminars experience suggested, the establishment of a European or an international program to build capacity by linking together senior social entrepreneurs from different countries, could help a lot seniors to develop social initiatives to address social needs in local communities. On the one hand, this international network would allow older people with different mindsets to assume a more open-minded attitude, with positive implications on the concrete experiences implemented by each other. On the other hand, the international experience in itself is appealing and stimulating for older people in terms of motivation, driving them to do their best in their respective local initiatives, to be then discussed in-depth in an international context. 

This qualitative study has several limitations. Even though it has been carried out in the local communities of different countries, its results certainly cannot claim for country generalization. The results of the study were highly influenced by the individual characteristics of the seniors, including their attitude towards the project. In some cases, bureaucratic tasks planned within the project, such as to write the individual portfolios or to fill-in templates for reporting the activities done, were considered by seniors as “time subtracted” from the concrete implementation of their initiatives, and some of them perceived these tasks as annoying. On the other hand, reporting was a crucial element to document the activity and the results of the project. In some cases, project partners helped seniors in collecting data and in reporting/assessing results. Another limitation concerns the impact assessment of the initiatives. Even if the initiatives implemented had the positive elements as explained above, more evidence would have been useful for evaluating more effectively their impact over time. Another aspect is, as is known, that older volunteers try to concentrate on the activities they enjoy more, and so prefer to act on their own terms [44]. This aspect was largely considered by the project partnership before the start of the project, and seniors were well informed about this. However, there have been some difficulties in this respect, together with some uncertainties in the initial phase of the project, about roles and responsibilities. In light of this, in order to avoid unexpected problems in this regard, professionals and researchers interested in implementing similar projects should consider the latter a very crucial aspect, and should dedicate particular efforts to explaining clearly and thoroughly to the seniors both the project and their expected tasks from the very beginning. 

Despite its many limitations, this study made it possible to increase evidence and knowledge concerning the beneficial role of older people acting as “social entrepreneurs” in European local communities. It also made it possible to activate socially innovative answers to social needs identified in local communities without any financial input from the project, thus making the initiatives implemented by the seniors sustainable, even after the end of the project. 

## 5. Conclusions

Building on the active aging framework, this study analyzed concrete experiences of older individuals acting as key players of social changes in six local communities of five European countries by engaging in volunteering activities. The results show that social solutions implemented by seniors to solve unmet needs, even involving other seniors and stakeholders through social brokering, seem to have produced a social value with a very low economic outflow, and, to different degrees, show encouraging results and impact. Since this “social experiment” provided evidence that senior social entrepreneuring could be a driver to solve societal problems, policy makers should sustain the spread of both social entrepreneurial mindset and practices with ad hoc measures at the European level, to catalyze the active potential of older people for the benefit of European local communities.

## Figures and Tables

**Table 1:** Sample description.

Local Community	N. Seniors	Gender	Age	Educational Level	Professional Status/ Background	ID
Aarhus (DK)	3	Male	62	High	Former top-level manager in health care, then freelance/semiretired and engaged in voluntary work in several organizations in his local community and at national level.	DK1
		Male	68	High	Former manager and consultant. After retirement, he has started an own business supporting older workers. He has also joined several networks of seniors and he volunteers.	DK2
		Female	68	High	Former upper secondary school teacher, retired. She volunteers in her city in different contexts (e.g., school, art museum, cultural activities).	DK3
Paris (FR)	3	Female	67	Intermediate	Former public servant and unionist, retired. Currently district councilor for an arrondissement of Paris and member of a NGO. Member of a choir, interested in helping seniors and being an active citizen.	FR1
		Female	71	High	Former journalist and director of English services in different organizations, currently responsible for an association of seniors and actively participating in an international senior-related organization.	FR2
		Female	67	Intermediate	Former secretary, after retirement fashion designer and organizer of workshops and conferences supporting seniors.	FR3
Pau (FR)	1	Female	60	Intermediate	From some years she has been working in community-based projects. Engaged in volunteering and in several initiatives and projects at the community level in different fields (e.g., social and economic solidarity, urban gardening, participatory housing).	FR4
Sabadell (ES)	4	Male	70	High	Before retirement, he was a bank office director and manager of private companies. Currently, he is doing voluntary work in an association where he supports small social companies and social entrepreneurs.	ES1
		Male	63	High	Former top manager of medium-large companies. Once retired, he engaged in voluntary work for a non-profit organization in which he shares his entrepreneurial and managerial knowledge and experience.	ES2
		Male	60	High	Retired. Former business manager and founder of an industrial company, he currently volunteers in a non-profit organization teaching economical and financial topics and supporting companies.	ES3
		Male	73	High	Retired. He worked for 40 years in the textile industry as an operational director, and then as a manger. Currently, he is a volunteer in a non-profit organization mentoring young people.	ES4
Shrewsbury (EN)	3	Female	50	Intermediate	Unemployed, professional experience in personal and nursing care, drug rehabilitation and education, engaged in community work for children and seniors.	EN1
		Male	85	High	Retired, professional experience in accountancy, civil service, insurance, pharmaceutical sales, organic food distribution, teacher, tattooing supplies. Engaged in community work.	EN2
		Male	57	High	Engaged in volunteering, unemployed. Experienced senior manager, involved in business development and change management.	EN3
Sofia (BG)	5	Female	60	High	Employed, University lecturer, member of the Bulgarian Nursing Association. Pensioner since the end of 2017.	BG1
		Female	60	High	Retired in 2017, former school director and English language teacher.	BG2
		Female	60	High	Employed, associate professor at the Institute of Neurobiology of the Bulgarian Academy of Sciences, head of Department of Sensory Neurobiology.	BG3
		Female	60	High	Pensioner since 2017, formerly employed as vice chairwoman of the Bulgarian Nurse Association, and as nurse at a hospital ward. Engaged in volunteering to support older people.	BG4
		Female	61	High	Employed as lawyer, volunteer experience, involved in different NGOs.	BG5

Educational level: High (ISCED 5+); Intermediate (ISCED 3-4); Low (ISCED 0-2). BG: Bulgaria; DK: Denmark; EN: England; ES: Spain; FR: France.

**Table 2:** Senior Social Entrepreneurship: description of the initiatives created by the seniors in the six European local communities.

Title	Local Community	Social Need(s)	Description of the Initiative	Social Innovation	Difficulties Encountered	Beneficiaries	ID
World Food Events	Aarhus (DK)	Lack of multicultural contacts.	Organization of “food events” (e.g., Indonesian, Portuguese, Peruvian food) aiming at favoring communication and reciprocal knowledge among Danish people and people of other nationalities, while eating food and receiving information about the nation from which the cooks come. The initiative, through the means of food, contributes to develop cultural exchange and social cohesion among people living in the community, in a soft and informal way.	5/5	None.	Around 25 people plus the cooks and their helpers.	DK1 DK2 DK3
Volunteers on a 1-day bus ride to the Island of Mors	Aarhus (DK)	To improve networking among volunteers.	The initiative aims at the empowerment of volunteers through the organization of a day trip for the creation of a network of volunteers living in a city and others living in a small rural community. Volunteers involved have shared experiences and got mutual inspiration for volunteering in their local communities.	3/5 (1, 2, 3)	Lack of resources for financing the activity (e.g., costs for the bus).	40 participants.	DK1 DK2 DK3
Music Band / Rock Band	Aarhus (DK)	Social isolation of disadvantaged people.	The initiative involves disadvantaged people (i.e., receivers of social pensions with mental and health problems) that meet each other weekly to play music. They also perform concerts for seniors living in nursing homes. The project aims and contributes to break social isolation and to develop confidence among the involved disadvantaged people, defined as a kind of “forgotten group” in the local community.	5/5	Some participants had to quit the initiative because of too much pressure and stress; difficulties to get funds for the equipment for playing.	Participants and spectators of concerts in nursing homes (some dozens, overall).	DK1
Breaking loneliness with music	Paris (FR)	Loneliness in a hospital environment.	The initiative consists in performing free vocal concerts through a choir, for the benefit of seniors living in health care facilities, with the aim of breaking their loneliness and to offer them moment of distraction, relax and happiness. Fifty volunteer singers were involved in the initiative.	4/5 (1, 2, 4, 5)	Administrative issues; ethical issues (for singing in hospitals); difficulty to recruit male singers; funding.	The number of spectators varied from concert to concert (dozens of individuals).	FR1
Seniors’ Empowerment Network	Paris (FR)	To strengthen social engagement of seniors.	Creation of a “special interest group” of seniors from different countries interested in social entrepreneuring based on an internet platform, with the aim of developing a wide network of seniors, empowering them and supporting their financial independence and social engagement.	4/5 (2, 3, 4, 5)	Too much time needed to nurture relationships among seniors.	Dozens of seniors from all over the word.	FR2
Express your creativity!	Paris (FR)	Social isolation and lack of self-confidence among retired people.	Organization of workshops and conferences for stimulating creativity, self-confidence and social networks among older people.	3/5 (1, 3, 5)	To find an appropriate location for the project activities.	25 individuals attend the workshops/conferences at a time.	FR3
Palanca Solidaire	Pau (FR)	Lack of multicultural integration.	“Palanca means leverage/help”. The initiative has various aims: to favor the participation of migrants in the economic exchange; make them feel useful knowing that they are not allowed to work until gained the refugee status; social inclusion by creating conditions for an exchange with the local population. Tools: exchange of services on a non-monetary base; membership in an association dealing with the help as godfather/godmother; exchanges of services based on the principle that 1 h of an offered service = 1 h of received service (time bank system).	5/5	Slowness of implementation; to find available migrants; language difficulties; To overcome intercultural differences.	12 individuals.	FR4
Helping social entities to improve their management	Sabadell (ES)	Social entities’ lack of knowledge about management, finance, labor aspects.	Offered knowledge and experience as CEO, financial directors, human resources directors, to social entities working in the local community (e.g., NGOs). First, a meeting with the entity is arranged. Once known the situation of the entity, a work plan is proposed. If the entity accepts to be helped, the team of seniors starts working for applying the proposed plan.	5/5	To get in touch with social entities; social entities’ difficulty to recognize to be in need of help.	Contacted 10-12 social entities. Help accepted by 3 social entities.	ES1 ES2 ES3
Workshops on entrepreneurship and business management in high schools	Sabadell (ES)	Lack of entrepreneurial attitude and of skills among young students.	The general aim of the initiative is to arrange workshops related to entrepreneurship and business management, in high schools. The initiative allows to learn and to get in touch with the current social and business reality. At the beginning, seniors, teachers, and students planned the topic they want to speak about, and then a schedule was arranged.	4/5 (1, 3, 4, 5)	Lack of social awareness about this topic; in some cases, difficulties to attract the attention of students.	9 workshops in different classes of 2 schools. 20-25 students involved in each workshop.	ES2 ES4
Market Drayton Community Enterprise	Shrewsbury (EN)	Urban and rural isolation.	Through a bus, a Community Hub (organizations as Diabetes UK, Samaritans, Macmillan Nursing, etc.) “is brought” to the urban and rural community by appointment. The community transport run by local volunteers picks up older and vulnerable people from their homes.	5/5	Funding; volunteer recruitment; lack of time; Managerial and administrative work/complexity.	Hundreds of people, mainly non-drivers, needing to travel to/from work, college, hospital.	EN1 EN2
Food Hub	Shrewsbury (EN)	Food Waste. Food Poverty. Social isolation.	The Food Hub initiative aims to collect food which is no longer marketable from supermarkets but is still safe to use and redistribute it to 40 charities and other not for profit groups in the local community. The initiative is focused in reducing food waste and in raising awareness on the issue.	5/5	Lack of a specialized project manager; difficulty to manage organization’s growth.	Approximately 750 people receive food from over 30 volunteers.	EN3
Social club “Academy 50+ and Friends”	Sofia (BG)	Lack of possibilities and facilities for social contacts and mutual aid among people living in a small local community.	Establishment of a social center with different activities and services (e.g., social and cultural exchange, measure of blood pressure, traditional folk crafts corner, etc.) where people living in a small village can have social contacts, talk to each other, to do different things together, to have fun and to help each other, in order to build a stronger local community.	4/5 (1, 2, 3, 4)	To find a right room for the social club and finances for the renovation of the room; little support from the mayor of the village.	More than 20 older individuals living in the village.	BG1
Give a Hand	Sofia (BG)	Isolation and lack of social life.	The initiative aims at: 1) creating a support team of neighbors who agreed to share their professional expertise and knowledge providing advices to other neighbors on some issues (e.g., law, care); 2) initiating “Coffee+”, a place where neighbors and friends can share their hobbies, to tackle isolation and promote an active and rewarding social life.	4/5 (1, 2, 3, 4)	Time management (i.e., difficulties in finding the right time for people to participate in the initiative).	About 30 individuals.	BG2
With care to the carers – Creation of an electronic library with free resources for health care professionals	Sofia (BG)	Lack of opportunities for nurses and caregivers to have access to new information about their job.	The initiative aims at favoring care professionals in obtaining new and updated knowledge through the creation of an electronic library with free resources, including materials in foreign languages. Initiative in an initial phase.	4/5 (1, 2, 3, 5)	Some technical problems linked to the software adopted.	NA, the initiative just started.	BG3
Basic computer skills for nurses and carers	Sofia (BG)	Lack of computer skills among nurses and caregivers aged 55+.	The initiative aims to train (in a friendly non-working environment, free of charge) some healthcare professionals (e.g., nurses) offering them basic computer skills since the latter are of growing importance for their job. Initiative still in a development phase.	2/5 (1, 3)	Busy work schedule of health care professionals and related lack of time.	NA, the initiative is going to start.	BG4
Happy gardeners	Sofia (BG)	Social isolation of older people.	The initiative aims to create an interest in working together among older people living in the same residential block, to improve their surroundings (e.g., taking care of green spaces), tackling in this way social isolation of older people.	2/5 (1, 3)	None.	34 individuals.	BG5

Social Innovation assessed according to the following Social Innovation Criteria: (1) relevance and consistency of social needs definition; (2) innovativeness and social acceptance of the solution; (3) openness of the innovation process (e.g., networking and stakeholder participation); (4) estimated impact of the solutions implemented; (5) potential capacity to implement, develop and maintain the initiative. Based on the assessment carried out by the project team, for each initiative, it is reported how many of the five criteria were fulfilled, and (in brackets) which of them. BG: Bulgaria; DK: Denmark; EN: England; ES: Spain; FR: France.
